# Deciphering the role of NtabCrRLK47 in rhizosphere microbiome remodeling and tobacco growth promotion

**DOI:** 10.3389/fpls.2026.1864982

**Published:** 2026-07-03

**Authors:** Guoping Wang, Yuqing Qi, Gaoyang Fei, Shibing Geng, Yunsong Chen, Canhua Lu, Moh Tariq, Yunye Zheng

**Affiliations:** 1 Yuxi Zhongyan Seed Co., Ltd, Yuxi, Yunnan, China; 2 Hunan Key Laboratory of Plant Functional Genomics and Developmental Regulation, College of Biology, Longping Agricultural College, Hunan University, Changsha, China; 3 Yunnan Academy of Tobacco Agricultural Sciences, Kunming, China

**Keywords:** CrRLK1L, NtabCrRLK47, plant growth, receptor-like kinases (RLKs), rhizosphere microbiome

## Abstract

Plant roots interact with diverse rhizosphere microbial communities that play essential roles in plant growth, nutrient acquisition, and stress adaptation. Although host genetics contributes to microbiome assembly, the molecular mechanisms governing microbial recruitment remain poorly understood. Members of the *Catharanthus roseus* receptor-like kinase 1-like (CrRLK1L) family regulate plant growth, immunity, and environmental responses, but their roles in rhizosphere microbiome regulation are largely unknown. Here, we investigated the function of NtabCrRLK47, a CrRLK1L receptor-like kinase in tobacco (*Nicotiana tabacum* L.), using CRISPR/Cas9-mediated gene knockout. *Ntabcrrlk47* mutants exhibited enhanced plant growth, including increased plant height, root length, and biomass, indicating that NtabCrRLK47 functions as a negative regulator of tobacco growth. Loss of NtabCrRLK47 significantly altered rhizosphere microbiome composition and function, increasing microbial diversity and enriching taxa such as *Myxococcota*, *Entotheonellaeota*, *Anaeromyxobacter*, *Aerococcus* and *Pseudomonas*, as well as functional genes associated with carbohydrate, amino acid, and energy metabolism. Two plant growth-promoting rhizobacteria, *Aerococcus urinaeequi* YX01 and *Pseudomonas koreensis* YX01, were isolated from the mutant rhizosphere. Both individual and combined inoculation significantly promoted tobacco growth, with co-inoculation showing a synergistic effect. Collectively, these findings demonstrate that NtabCrRLK47 acts as a negative regulator of tobacco growth and influences rhizosphere microbiome-associated growth responses through the recruitment of beneficial microorganisms. These results provide new insights into CrRLK1L-mediated host–microbiome interactions and identify two promising PGPR strains for microbiome-assisted crop improvement.

## Introduction

Tobacco (*Nicotiana tabacum* L.) is a key global crop, covering 1 million hectares in 2023. However, unsustainable agricultural practices have led to soil degradation, limiting plant growth, increasing disease susceptibility, and reducing leaf quality (Zhang, 2024; Fang et al., 2023; Niu et al., 2025). The root-associated microbiome, including the rhizosphere and endosphere, plays a crucial role in plant health by regulating key physiological and defense processes. Microbes in this niche enhance plant growth through phytohormone modulation (Lugtenberg and Kamilova, 2009), nutrient mobilization (Fabiańska et al., 2019), pathogen antagonism (Durán et al., 2018), immune regulation (Pieterse et al., 2014), and stress tolerance (Xu and Coleman-Derr, 2019). Soil properties significantly shape microbiome composition, influencing nutrient uptake and plant adaptation to stress, such as drought and salinity (Xu et al., 2021; Li et al., 2021). The root microbiome is often referred to as the plant’s “second genome” due to its integration with plant physiology (Berendsen et al., 2012). Root-associated microbes also mediate induced systemic resistance (ISR), priming plants for enhanced immune responses (Zamioudis and Pieterse, 2012). *Pseudomonas fluorescens* WCS417 is a well-studied ISR inducer, suppressing flagellin-triggered immunity in *Arabidopsis thaliana* through the secretion of low-molecular-weight molecules (Millet et al., 2010). Plant growth-promoting rhizobacteria (PGPR) with ACC deaminase reduce ethylene accumulation under stress, improving plant growth and stress tolerance (Gupta and Pandey, 2019). *Streptomyces* spp. contributes to plant health by producing bioactive metabolites that promote growth, immunity, and soil nutrient dynamics (Viaene et al., 2016; Olanrewaju and Babalola, 2019). Root-associated microbial communities, including bacteria, archaea, fungi, and oomycetes, are critical for stress tolerance and ecosystem functions such as nitrogen fixation and biocontrol of root pathogens (Danish et al., 2020). Beneficial microbes have been shown to enhance plant growth under water-limited conditions in various crops, including foxtail millet (Niu et al., 2018), maize (Sandhya et al., 2010), wheat (Meenakshi et al., 2019), and tomato (Gowtham et al., 2020).

The assembly and function of the root microbiome are governed by complex interactions between host plants and their environment, with plant genetic factors acting as central regulators of microbial community structure (Trivedi et al., 2020). Root exudates and plant immune responses are key modulators of the rhizosphere, influencing microbial recruitment, colonization, and activity (Bai et al., 2022). Plant genetic factors exert “top-down” control over rhizosphere microbial communities through molecular signaling networks (Shi et al., 2020). For instance, modulation of nutrient-uptake genes enables plants to recruit beneficial microbes that enhance nutrient stress tolerance (Teixeira et al., 2019; Zhang et al., 2019). This process is mediated by changes in root architecture—such as increased surface area, lateral root development, and root hair formation—which reshape the spatial and chemical environment of the rhizosphere (Yu et al., 2021). Developmental regulators such as *AXR2* and *RHD6* in *A. thaliana* alter root hair formation and exudate profiles, impacting microbial assemblages (Yi et al., 2010). Similarly, maize genes including *RTH6*, *LRT1*, *RUM1*, and *RTC* influence root development and associated microbial communities (Coudert et al., 2010). Nutrient transporter genes such as *NRT1.1* and *PHT1* facilitate the recruitment of nitrogen-fixing and phosphate-solubilizing microbes, reshaping microbiome composition (Léran et al., 2013; Chien et al., 2022). Secondary metabolic pathways, including flavonoid biosynthesis genes (*FNS*, *C2*) and *ABC* transporters, further determine microbial diversity and taxa composition (Falcone Ferreyra et al., 2015; Cox et al., 2019). Defense-related genes also modulate the rhizosphere: pattern-recognition receptors (PRRs) (e.g., *FLS2*) and NLR proteins detect microbial signals and activate immune responses that alter nutrient and metabolite availability (Chen et al., 2020; Escudero-Martinez et al., 2023). Therefore, host genetics coordinates these outcomes through integrated regulation of root architecture, exudation, immunity, and nutrient acquisition pathways (Wang et al., 2022).

Among molecular regulators, receptor-like kinases (RLKs) play a crucial role in perceiving extracellular cues and activating defense and developmental pathways that shape root-associated microbiota (Dievart et al., 2020; Wang et al., 2020). PRRs detect microbe-associated molecular patterns (MAMPs) and modulate microbial access at the root–soil interface. For example, the flagellin receptor FLS2 influences rhizosphere β-diversity, and *fls2* mutants exhibit altered bacterial community composition, underscoring the role of immune surveillance in microbiome structuring (Fonseca et al., 2022). However, not all PRRs exert similar effects; *efr* and *cerk1* display limited microbiome shifts, indicating functional specialization among PRR pathways (Fonseca et al., 2022). A well-characterized example is the CrRLK1L member *FERONIA* (*FER*), which integrates peptide signaling, cell wall integrity, and immune regulation to control root colonization. *fer* mutants host distinct rhizosphere microbiomes enriched in *Pseudomonas*, linked to reduced apoplastic ROS and altered root surface redox status (Song et al., 2021). FER interacts with RALF peptides and cell wall sensors, modulating plasma membrane nanodomain organization, receptor mobility, and pattern-triggered immunity thereby influencing microbial recruitment (Xie et al., 2022). Despite growing insights in the microbiome regulation by RLKs, it remains largely unknown if specific microbes shaped by RLKs could regulate plant growth.

Despite increasing evidence that receptor-like kinases such as FER influence rhizosphere microbiome assembly, it remains unclear whether other members of the CrRLK1L family similarly regulate microbial recruitment and whether microbiome remodeling contributes to RLK-mediated plant growth regulation (Song et al., 2021; Tang et al., 2022; Galindo-Trigo et al., 2016). In particular, the biological function of NtCrRLK in tobacco and its potential role in shaping rhizosphere microbial communities have not been investigated. We hypothesized that *NtabCrRLK47* acts as a regulator of plant growth by influencing rhizosphere microbiome composition and the recruitment of beneficial microorganisms. To test this hypothesis, we generated CRISPR/Cas9-mediated *NtabCrRLK47* knockout mutants and characterized their growth phenotypes, rhizosphere microbial communities, and microbial functional profiles using 16S rRNA amplicon sequencing and metagenomic analyses. Furthermore, we isolated and characterized bacterial strains enriched in the mutant rhizosphere and evaluated their growth-promoting effects on tobacco. This study provides new insights into the role of CrRLK1L-mediated host–microbiome interactions and identifies NtabCrRLK47 as a potential regulator of microbiome assembly and plant growth.

## Materials and methods

### Gene editing of *NtabCrRLK47*

Candidate guide RNA (gRNA) target sites were designed based on the coding sequences (CDS) of *NtabCrRLK47*. gRNA evaluation was performed primarily using two online platforms (CRISPRdirect: https://crispr.dbcls.jp/ and CCTop: https://cctop.cos.uni-heidelberg.de/), and final selections were made based on integrated off-target and efficiency scoring from both tools. To enhance gene knockout efficiency, 2 gRNAs were selected for one gene. CRISPR-Cas9 vector assembly was carried out using the binary genome editing backbone pHSbdcas9i. For each selected target, primers were designed following parameter optimization, and synthetic target fragments were ligated into the vector. Recombinant clones were validated and subsequently used for *Agrobacterium*-mediated transformation.

### Growth phenotype analysis of *Ntabcrrlk47* mutants

Seeds of homozygous *Ntabcrrlk47* mutant tobacco and WT controls were sown in nutrient pots containing 110 g of soil substrate. Plants were grown under controlled conditions of (26 ± 2) °C with a 16 h light/8 h dark photoperiod. After 7 days of germination, seedlings were thinned to retain one plant per pot. Growth was continued for 4 weeks, with appropriate watering maintained throughout the cultivation period. At the end of the growth stage fresh weight, leaf area, root length and other growth parameters were measured and recorded. All growth assays were conducted using 3 biological replicates per genotype and were repeated in 3 independent experiments. Comparative analysis between mutants and wild-type plants was performed to assess the impact of *Ntabcrrlk47* mutations on tobacco growth and statistical evaluation of the growth phenotypes was conducted.

### 16S rRNA gene sequencing and library construction

Genomic DNA of the samples was extracted using the MagPure Soil DNA LQ Kit (Magan) following the manufacturer’s instructions. DNA quantification and purity assessment were subsequently performed to ensure the smooth implementation of subsequent amplification experiments. The 16S rRNA gene flanks with conserved primer-binding regions, and harbors nine hypervariable regions (V1–V9) in between. The universal primers 27F and 1492R were employed for gene amplification. The polymerase chain reaction (PCR) system contained approximately 1–10 ng of template DNA, 0.5 μM of each primer, and standard PCR premix. The PCR cycling conditions were set as follows: initial denaturation at 94 °C for 2 min; followed by 25–35 cycles of denaturation at 94 °C for 30 s, annealing at 50–60 °C for 30 s, and extension at 72 °C for 1 min; and a final extension step at 72 °C for 5 min. The target amplicon with a length of approximately 1.4–1.5 kb was thus obtained. The quality of amplicons was evaluated via agarose gel electrophoresis. PCR products were then purified using AMPure XP beads (Agencourt, Beckman Coulter) and subjected to a second round of PCR amplification. After the second round of purification with AMPure XP beads, the final amplicons were quantified using the Qubit dsDNA Assay Kit (Thermo Fisher Scientific, USA), and their concentrations were adjusted to meet the sequencing requirements. Sequencing was performed by OE Biotech Co., Ltd. (Shanghai, China) on the Illumina NovaSeq 6000 platform using the 250 bp paired-end sequencing mode.

### Metagenomic DNA extraction, library construction, and bioinformatic analysis

Total microbial DNA was extracted from samples using the QIAamp^®^ Fast DNA Stool Mini Kit. After quantifying DNA concentration and assessing purity with a NanoDrop 2000 spectrophotometer, and evaluating DNA integrity via 1% agarose gel electrophoresis, DNA fragmentation was performed using S220 Focused-ultrasonicators. The fragmented DNA was purified with Agencourt AMPure XP beads, followed by metagenomic library construction using the TruSeq Nano DNA LT Sample Preparation Kit. After library quality validation, paired-end sequencing with a read length of 150 bp was conducted on the Illumina NovaSeq 6000 platform. Metagenomic library construction, sequencing, and subsequent bioinformatic analyses were performed with the assistance of OE Biotech Co., Ltd. (Shanghai, China). Raw sequencing data were processed as follows: low-quality reads and adapter sequences were filtered out using Trimmomatic, and host-derived sequences were removed via bowtie2. *De novo* metagenomic assembly was then performed using MEGAHIT, and open reading frames (ORFs) were predicted with Prodigal and translated into amino acid sequences (Li et al., 2015). A non-redundant gene catalog was constructed using CD-HIT, and gene abundance quantification was accomplished by mapping reads to the catalog with bowtie2.

### Functional annotation and enrichment analysis

To characterize the functional potential of the microbial communities, predicted non-redundant genes were functionally annotated against the KEGG database using DIAMOND with an E-value threshold of 1 × 10^−5^ (Buchfink et al., 2015). KEGG Orthology (KO) assignments were used to reconstruct metabolic pathways, and pathway abundances were calculated by summing the abundances of genes assigned to each KO. Differentially enriched KEGG pathways among treatments were identified based on normalized pathway abundance profiles using one-way ANOVA followed by Tukey’s multiple comparison test. Pathways with P < 0.05 were considered significantly enriched.

For carbohydrate-active enzyme (CAZyme) analysis, predicted protein sequences were searched against the CAZy database using hmmscan implemented in HMMER (v3.3). CAZyme family abundance was estimated according to the abundance of annotated genes. Differential enrichment of CAZyme families among groups was evaluated using one-way ANOVA, and CAZyme families with P < 0.05 were considered significantly enriched.

Linear discriminant analysis effect size (LEfSe) was employed to identify microbial taxa and functional features significantly associated with different treatments. LEfSe analysis was performed using the Galaxy LEfSe pipeline, applying the non-parametric Kruskal–Wallis test for group comparisons and linear discriminant analysis (LDA) for effect size estimation. Features with a Kruskal–Wallis P value < 0.05 and an LDA score > 2.0 were considered significant biomarkers. Principal component analysis (PCA), principal coordinate analysis (PCoA), and additional statistical analyses were conducted using R software, and all statistical tests were considered significant at P < 0.05.

### Isolation, screening and identification of root-associated bacteria

Root-associated bacterial communities were isolated from four-week-old *NtabCrRLK47* mutant tobacco plants. Following removal of loosely attached soil, roots were washed with sterile water and homogenized in sterile phosphate-buffered saline. The resulting root slurry was serially diluted and subjected to dilution-to-extinction cultivation in 96-well plates containing nutrient medium. After incubation at 28 °C, wells exhibiting bacterial growth were streaked onto solid medium to obtain pure colonies. Purified isolates were preserved in 50% glycerol stocks at −80 °C.

To identify plant growth-promoting bacteria, individual isolates were cultured and evaluated using tobacco seedling inoculation assays. Growth-promoting activity was assessed by comparing shoot growth, root length, leaf area, and fresh biomass of inoculated plants with uninoculated controls. Isolates showing reproducible and significant growth-promoting effects across independent experiments were selected for further characterization. Two representative isolates exhibiting the strongest growth-promoting effects were designated YX01 strains.

Species identification was performed by amplification of the nearly full-length 16S rRNA gene using universal primers 27F and 1492R. PCR products were sequenced, and the resulting sequences were compared against the NCBI nucleotide database using BLAST. Based on sequence similarity analysis, the isolates were identified as *Aerococcus urinaeequi* YX01 and *Pseudomonas koreensis* YX01.

### Bacterial inoculum preparation and plant inoculation

For inoculum preparation, bacterial isolates were cultured overnight in TSB medium at 28 °C with shaking. Cells were harvested by centrifugation at 5000 rpm for 3 min, washed twice with sterile distilled water, and resuspended in sterile distilled water. The bacterial suspension was adjusted to an optical density of OD600 = 0.1 using a biophotometer.

Tobacco seedlings were germinated for one week and subsequently transplanted into individual pots containing soil. After two weeks of growth, each plant was inoculated with 5 mL of bacterial suspension (OD600 = 0.1) by soil drench application. A second inoculation using the same procedure was performed one week later. Control plants received an equal volume of sterile distilled water. After 2 weeks, the final growth-promoting effect of the bacterial microbes on the plants is assessed. Each treatment consisted of 3 biological replicates and the experiment was repeated independently 3 times.

### Statistical analysis

Statistical significance was determined by conducting Bartlett’s test or one-way ANOVA using GraphPad 9 software. Statistical significance between two groups was assessed using Bartlett’s test, whereas comparisons among multiple treatments were performed using one-way analysis of variance (ANOVA) followed by Dunnett’s multiple comparison test. Differences were considered statistically significant at *P* < 0.05.

## Results

### Mutation of NtabCrRLK47 has positive impact on tobacco growth

**Figure 1:**
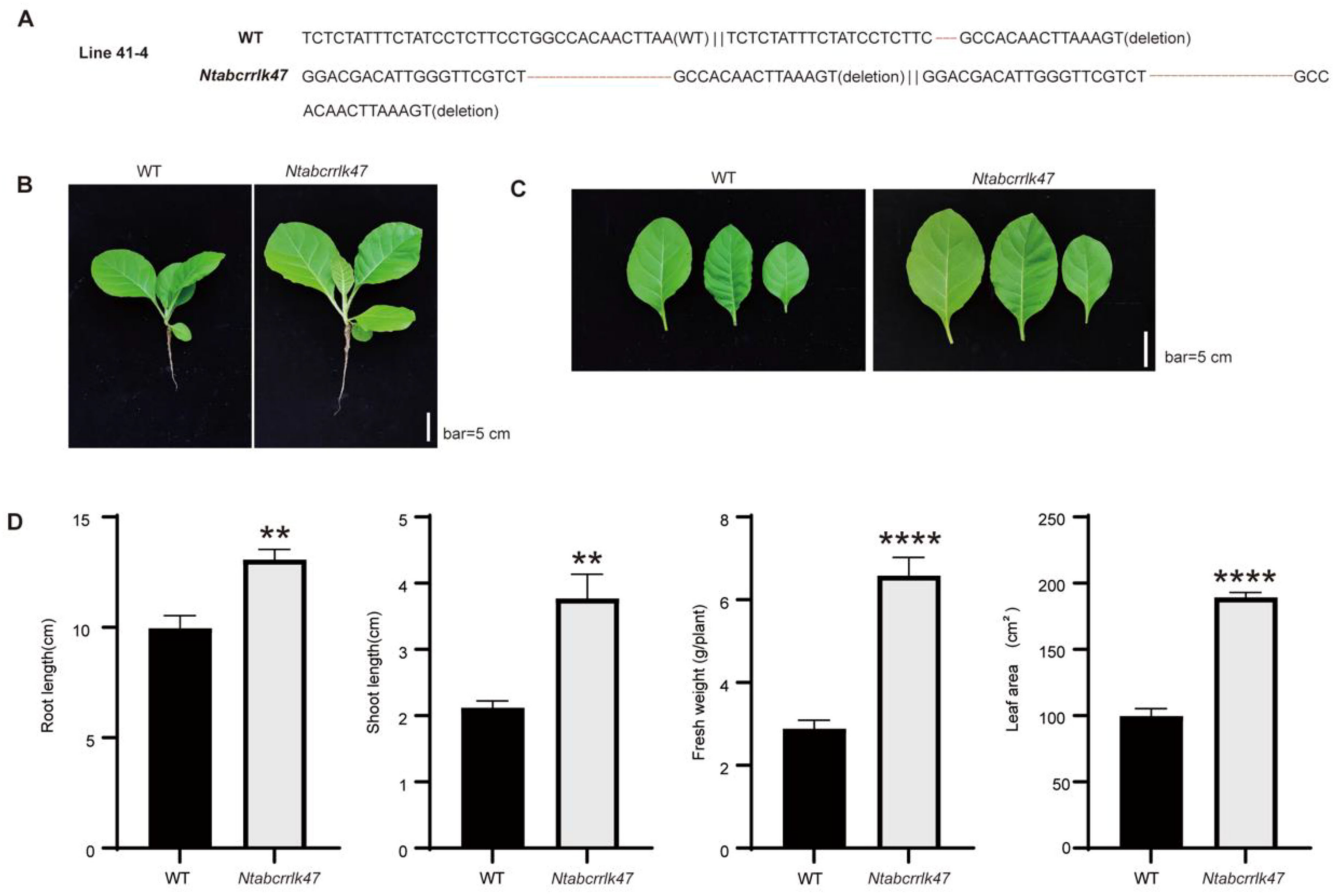
Mutation of *NtabCrRLK47* enhances tobacco growth. **(A)** Illustration of the *Ntabcrrlk47* mutant line, highlighting its distinct characteristics compared with the wild-type (WT) plants. **(B)** Representative images comparing plant height and primary root length between *Ntabcrrlk47* and WT plants. **(C)** Representative images showing leaf area in *Ntabcrrlk47* and WT plants. **(D)** Quantitative analysis of growth parameters showing differences between *Ntabcrrlk47* and WT plants. Values represent means ± SD (n = 4 biological replicates). Statistical significance was determined by one-way ANOVA; asterisks indicate significant differences (**p < 0.01; ****p < 0.001). The experiment was independently repeated three times with similar results.

To dissect the biological function of *NtabCrRLK47* in tobacco, we generated a knockout mutant of *NtabCrRLK47* in Yunyan 87—an elite tobacco cultivar widely grown in Southern China. The mutant line (#4) harbored a 300 bp deletion within the CDS of *NtabCrRLK4*7, leading to the disruption of gene function (**Figure 1A**). Phenotypic characterization under controlled growth chamber conditions revealed that *NtabCrRLK47* knockout plants exhibited enhanced growth performance compared to WT counterparts (**Figures 1B, C**). Quantitative analysis confirmed that key agronomic traits, including plant height, root length, leaf area and fresh weights of aboveground and underground tissues, were significantly increased in the *NtabCrRLK47* mutant relative to WT (**Figure 1D**). Collectively, these findings demonstrate that *NtabCrRLK47* functions as a negative regulator of tobacco growth and its loss-of-function mutation confers a growth-promoting phenotype.

### Mutation of *NtabCrRLK47* enhances microbial diversity in tobacco rhizosphere

**Figure 2:**
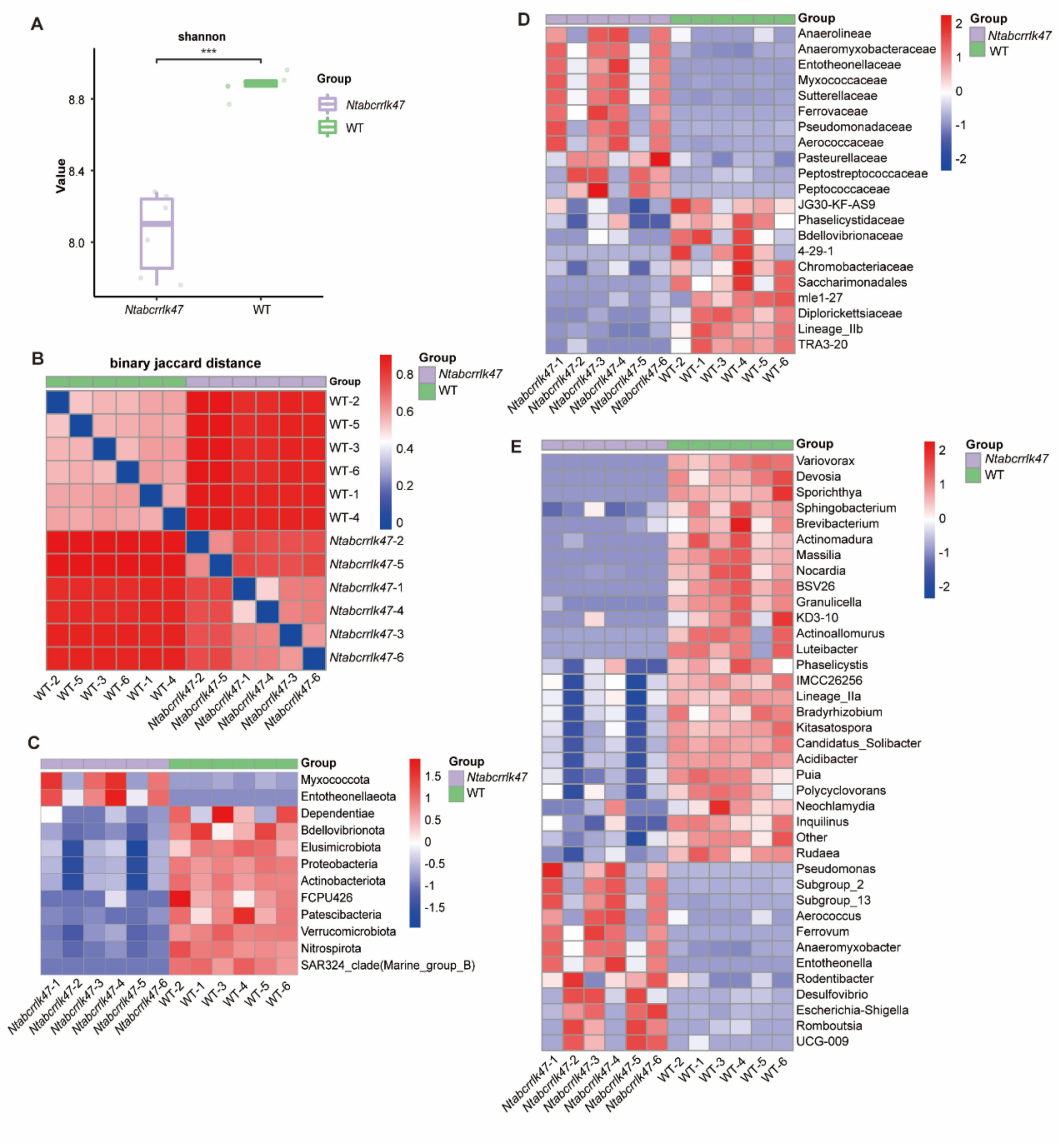
Mutation of *NtabCrRLK47* alters microbial diversity in the tobacco rhizosphere. **(A)** Alpha diversity, estimated the Shannon index, showing a significant difference in microbial diversity between the *Ntabcrrlk47* mutant and WT rhizosphere (***p < 0.001). **(B)** Binary Jaccard distance heatmap illustrating differences in microbial community composition, with clear clustering and separation between *Ntabcrrlk47* and WT samples. **(C)** Heatmap showing the relative abundances of dominant bacterial phyla across the experimental groups. **(D)** Differential abundance patterns of bacterial families between *Ntabcrrlk47* and WT samples. **(E)** Heatmap of bacterial genera showing taxa enriched or depleted in *Ntabcrrlk47* relative to WT.

The plant rhizosphere microbiome, as a pivotal “functional partner” in host growth and development, exerts core regulatory effects on nutrient acquisition and stress responses (Araujo et al., 2025). To elucidate the regulatory role of the RLK gene *NtabCrRLK47* in the assembly of tobacco root microbiome, we systematically characterized the rhizosphere microbial communities of *Ntabcrrlk47* mutants and WT tobacco plants at the tillering stage. A total of 12 samples, including 6 biological replicates per genotype, were subjected to 16S rRNA gene V3-V4 region amplicon sequencing using the Illumina MiSeq platform. Sequencing data were processed through standardized quality control pipelines, including Trimmomatic-based quality filtering (Q30 ≥ 90%), DADA2-mediated denoising, and chimera removal. The valid read pass rate reached 95.38%–95.75%, with a read merging efficiency of 69.74%–77.07% and a non-chimeric sequence ratio of 63.92%–69.91%, ensuring high data reliability. A total of 5,230 Amplicon Sequence Variants (ASVs) were detected across all samples (**Supplementary Table 1**), indicating a stable core microbial reservoir in tobacco roots. However, significant inter-group differences in ASV abundance were observed: the WT sample WT-1 exhibited high diversity (1,222 ASVs), whereas the mutant sample *Ntabcrrlk47–*1 showed reduced diversity (679 ASVs). Microbiome diversity analysis using QIIME2 revealed that the *Ntabcrrlk47* mutant had a significantly lower Shannon index than WT (p < 0.001, Wilcoxon signed-rank test) (**Figure 2A**). Principal Coordinate Analysis (PCoA) based on Bray-Curtis dissimilarity demonstrated distinct clustering of mutant and WT samples (**Figure 2B**), and PERMANOVA analysis confirmed statistically significant differences in community structure (R² = 0.32, p = 0.02).

Taxonomic composition analysis showed phylum-level disparities: the relative abundances of *Myxococcota* and *Entotheonellaeota* in the mutant were increased by 42.3% and 38.7% compared to WT, respectively, while *Proteobacteria* (35.2%) and *Actinobacteria* (28.6%) were more abundant in WT (**Figure 2C**). At the family level, *Myxococcaceae*, *Aerococcaceae*, *Entotheonellaceae* and *Pseudomonadaceae* were specifically enriched in the mutant, whereas *Phaselicystidaceae* and *Diplorickettsiaceae* dominated the WT (**Figure 2D**). Differential abundance analysis at the genus level (LDA > 4, p < 0.05) identified *Anaeromyxobacter* and *Entotheonella* as the most significantly enriched genera in the mutant, with relative abundances 3.2-fold and 2.8-fold higher than those in WT, respectively. The genera *Aerococcus* and *Pseudomonas* were detected in both WT and *Ntabcrrlk47* rhizosphere microbiomes; however, their relative abundance was markedly higher in the mutant plants (**Figure 2E**). Combined with Wilcoxon rank-sum test and t-test results (**Supplementary Figures 1A–D**), our study confirms that *NtabCrRLK47* mutation reshapes the tobacco root microbiome by significantly increased α-diversity, altering β-diversity characteristics, and specifically enriching functional taxa such as *Myxococcota*, *Entotheonellaeota*. Given that these enriched taxa often possess nitrogen-fixing, phosphate-solubilizing, and plant-growth-promoting capabilities, we hypothesize that *NtabCrRLK47* may regulate the rhizosphere microenvironment to recruit functional microbes, thereby enhancing plant growth vitality and stress resilience. These findings provide a core basis for deciphering the molecular mechanisms underlying host-microbiome interactions.

### *Ntabcrrlk47* enriches rhizosphere microbial genes for adaptation, metabolism, and antibiotic resistance in tobacco

To systematically decipher the molecular mechanisms by which the *Ntabcrrlk47* mutation regulates the assembly and function of the tobacco rhizosphere microbiome, we performed metagenomic sequencing on rhizosphere samples from *Ntabcrrlk47* mutants and WT tobacco plants, with three biological replicates per group. Integrated bioinformatic analyses encompassing taxonomic annotation, gene expression quantification, and functional pathway enrichment confirmed that *NtabCrRLK47* serves as a key hub gene governing the rhizosphere microbiome.

**Figure 3:**
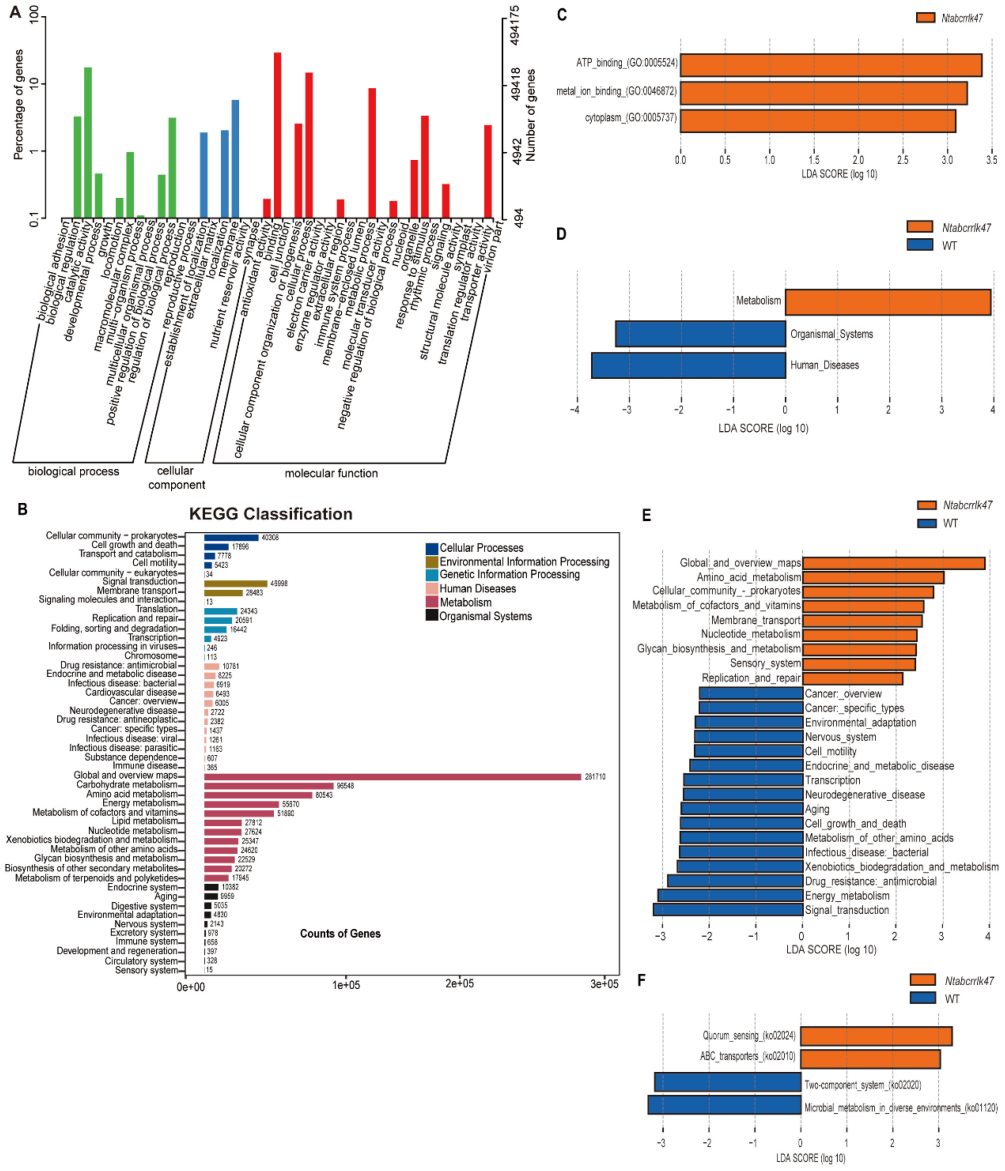
*Ntabcrrlk47* enriches rhizosphere microbial genes associated with adaptation, metabolism and antibiotic resistance in tobacco. **(A)** Distribution of gene counts across Gene Ontology (GO) categories, including biological process, cellular component and molecular function. **(B)** KEGG functional classification showing gene counts across major metabolic and biochemical pathways. **(C)** Linear Discriminant Analysis (LDA) scores (log10) for selected GO including ATP binding, metal ion binding, cytoplasm, enriched in the *Ntabcrrlk47* rhizosphere. **(D)** LDA comparison of broad functional categories, showing increased representation of metabolic processes and reduced representation of organismal systems in *Ntabcrrlk47* relative to WT. **(E)** LDA scores for key KEGG pathways, including global and overview maps and protein metabolism. **(F)** LDA scores for signaling-related pathways, highlighting the enrichment of quorum sensing, ABC transporters, and two-component systems in *Ntabcrrlk47*.

Gene Ontology (GO) enrichment analysis demonstrated that rhizosphere microorganisms in the *Ntabcrrlk47* mutant exhibited a prominent enrichment signature in biological processes tightly linked to adaptive traits, including biological adhesion, regulatory processes, catalytic activity, and developmental processes (**Figure 3A**). These functional modules constitute the molecular underpinnings for mediating dynamic plant-rhizosphere microbial interactions, and their enrichment implies the formation of a more active microecological interaction network in the mutant rhizosphere. Notably, genes affiliated with the microbial taxa *Anaeromyxobacter* and *Entotheonella* were highly enriched in the *Ntabcrrlk47* mutant, suggesting their potential roles in facilitating beneficial plant-microbe interactions. At the molecular function level, genes associated with ATP binding, metal ion binding, and cytoplasmic localization were highly enriched in the rhizosphere microorganisms of *Ntabcrrlk47* mutants (**Figure 3C**) functions critical for microorganisms to maintain energy metabolism, ion homeostasis, and core physiological activities under stressful conditions, thereby directly enhancing the environmental tolerance and survival competitiveness of rhizosphere microbiota.

Kyoto Encyclopedia of Genes and Genomes (KEGG) pathway enrichment analysis demonstrated that genes involved in metabolic pathways were significantly upregulated in the rhizosphere microbiota of *Ntabcrrlk47* mutants, encompassing carbohydrate metabolism (e.g., glycogen biosynthesis), amino acid catabolism and energy production-related pathways (**Figure 3B**). Concomitantly, the expression levels of pathways regulating membrane transport and xenobiotic degradation were coordinately elevated—a feature indicative of substantially enhanced nutrient acquisition efficiency and detoxification capacity of harmful substances in the mutant rhizosphere microbiota under stress conditions (**Figure 3B**). Linear Discriminant Analysis Effect Size (LEfSe) comparison at KEGG Level 1 revealed that metabolic pathways exhibited a particularly prominent enrichment signature in *Ntabcrrlk47* mutants relative to WT (**Figure 3D**). At KEGG Level 2, specific enrichment was observed in pathways associated with amino acid metabolism, cofactor biosynthesis, and glycan biosynthesis—key processes underlying microbial intercellular signaling, environmental adaptation, and biofilm formation (**Figure 3E**). At KEGG Level 3, the enrichment signature of genes related to ABC transporters (ko02010) and quorum sensing (ko02024) was remarkably pronounced (**Figure 3F**), suggesting that the *Ntabcrrlk47* mutation enhances synergistic interactions and nutrient uptake efficiency among rhizosphere microorganisms.

**Figure 4:**
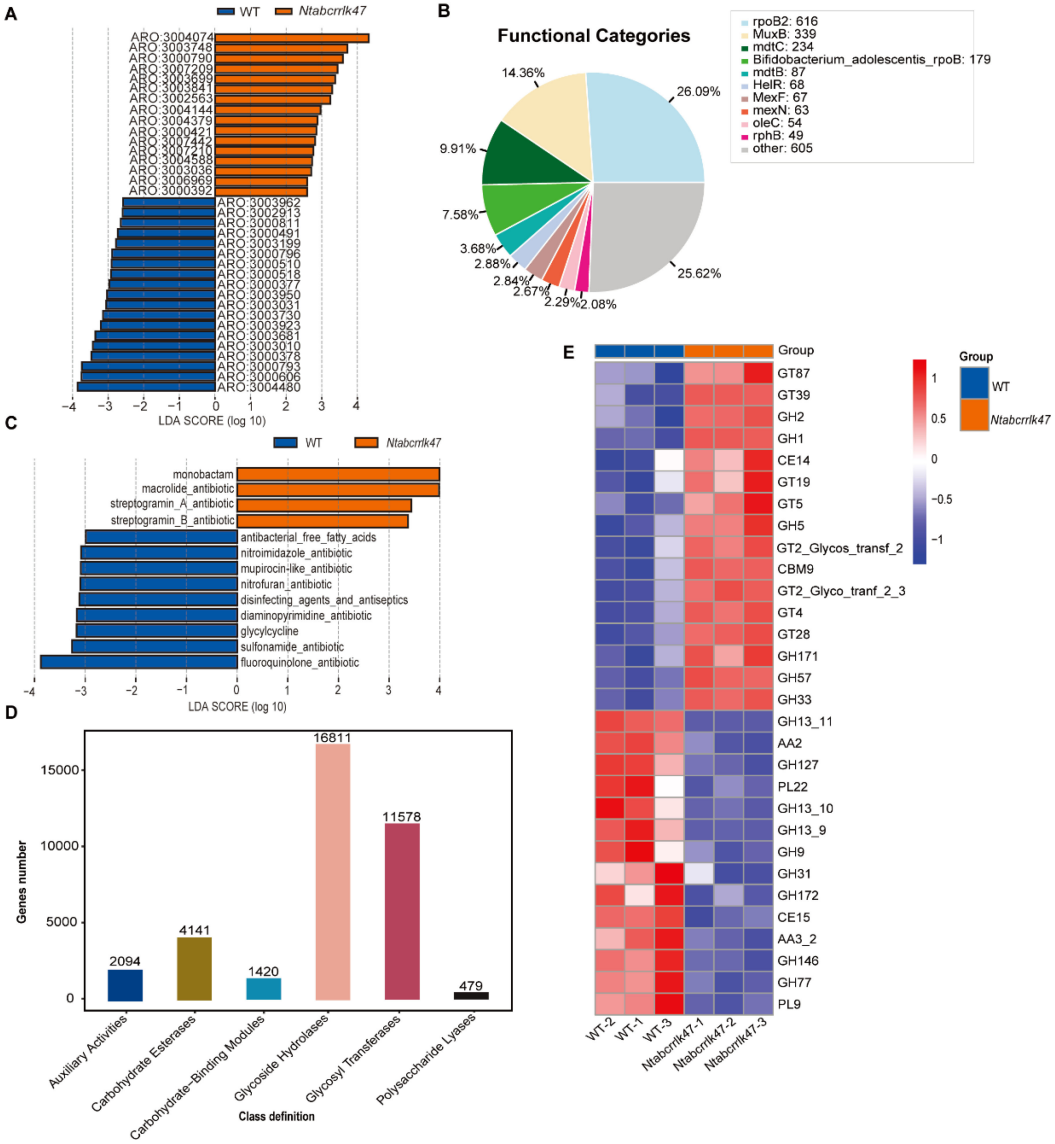
Differential microbial and functional gene profiling between WT and *Ntabcrrlk47*. **(A)** Linear Discriminant Analysis (LDA) scores of differentially abundant Antibiotic Resistance Ontology (ARO) terms, highlighting features enriched in WT and *Ntabcrrlk47*. **(B)** Pie chart showing the distribution of major functional gene categories across samples, with *rpoB2* and *MuxB* among the dominant categories. **(C)** LDA scores (log10) for antibiotic resistance–related functional categories, showing enrichment of monobactam, macrolide, and streptogramin resistance genes in *Ntabcrrlk47* relative to WT. **(D)** Abundance of carbohydrate-active enzyme (CAZy) classes, including auxiliary activities (AAs), carbohydrate esterases (CEs), carbohydrate-binding modules (CBMs), glycoside hydrolases (GHs), glycosyltransferases (GTs), and polysaccharide lyases (PLs). **(E)** Heatmap of differentially abundant CAZy families across samples, clearly distinguishing WT and *Ntabcrrlk47*.

Functional annotation based on Clusters of Orthologous Groups (KO) further validated the enrichment of genes involved in quorum sensing, ABC transporters, cofactor biosynthesis, fatty acid metabolism, and nucleotide sugar biosynthesis (**Supplementary Figure 2**) (Powell et al., 2014). These pathways synergistically enhance microbial energy generation and metabolic homeostasis, thereby augmenting microbial adaptive performance under stress conditions. Comprehensive Antibiotic Resistance Database (CARD) analysis revealed a significantly higher abundance of antibiotic resistance genes (ARGs) in the *Ntabcrrlk47* mutant, with prominent enrichment of resistance markers for macrolides and monobactams (e.g., ARO:3004074, ARO:3003748) (**Figures 4A, C**)—a finding indicative of a more stress-resilient rhizosphere microbiome. Concomitantly, the enrichment of multidrug efflux system-related genes (e.g., *rpoB2*, *MuxB*, *mdtC*) reinforces microbial antimicrobial resistance and optimizes stress response signaling cascades (**Figure 4B**).

Carbohydrate-Active enZymes (CAZy) annotation analysis revealed that genes involved in complex carbohydrate degradation were significantly enriched in the rhizosphere microbes of *Ntabcrrlk47* mutants, with the most substantial increase in the abundance of genes belonging to the Glycoside Hydrolases (GH) and Glycosyltransferases (GT) families (**Figures 4D, E**; **Supplementary Figures 3A–D**). These enzymes efficiently degrade complex carbohydrates in the rhizosphere to provide sufficient carbon sources and energy for microbial growth, reshaping the rhizosphere microbial community structure while indirectly improving plant health and stress tolerance. Collectively, the *Ntabcrrlk47* mutation drives the formation of a rhizosphere microbiome with augmented microbial adaptability, metabolic versatility, and antibiotic resistance, optimizing the plant-microbe interaction network and offering substantial potential for agricultural applications aimed at improving crop stress resilience and productivity.

### Isolation and identification of growth-promoting microbes from *Ntabcrrlk47* rhizosphere

To identify the microbial taxa underlying the enhanced growth phenotype of *Ntabcrrlk47* mutant tobacco, we first isolated 500 associated bacterial strains from its rhizosphere. To streamline the screening workflow, we randomly pooled every 5 isolates into a single group in subsequent experiments, ultimately classifying these bacteria into 100 independent groups based on morphological characteristics (**Supplementary Table 2**). Under artificially controlled environmental conditions, a standardized tobacco growth assay system was employed to screen the growth-promoting capacity of all groups. Results showed that Group #80 exhibited consistent and significant activity in promoting both shoot and root growth of tobacco (**Supplementary Figure 4A**).

To identify the specific microbes exerting the core growth-promoting effect within this group, Group #80 was further subdivided into 5 individual strains, designated as A3, C9, C8, D8, and A12. The function of each individual strain was independently validated through triplicated growth-promotion assays. Notably, strains A12 and C8 displayed the most prominent growth-promoting effects, with both significantly increasing tobacco biomass, root length, and shoot development (**Supplementary Figures 4B, C**). Subsequently, we performed molecular biological identification of strains A12 and C8. Genomic DNA was extracted from strains A12 and C8, and the full-length 16S rRNA gene was amplified by PCR using the universal primers 27F/1492R. The PCR products were verified by electrophoresis, purified, and subjected to Sanger sequencing. High-quality sequences were aligned against the Silva 138 and NCBI GenBank databases, with initial species-level classification based on a sequence similarity threshold of ≥98.7% (**Supplementary Figures 4D, E**). Phylogenetic trees were constructed using the neighbor-joining method to validate their taxonomic positions. The result revealed that strain A12 was identified as *A. urinaeequi* YX01 (phylum *Firmicutes*/family *Aerococcaceae*). Strain C8 was classified as *P.koreensis* YX01 (phylum *Pseudomonadota*/family *Pseudomonadaceae*). Collectively, these findings indicate that *A. urinaeequi* YX01and *P. koreensis* YX01 are the key functional taxa underlying the growth-promoting phenotype observed in the rhizosphere of *Ntabcrrlk47* mutants. Their presence likely reflects the remodeling of beneficial rhizosphere microbial communities mediated by the knockout of the *NtabCrRLK47* gene.

### Effects of individual and combined inoculation of *A. urinaeequi* YX01 and *P. koreensis* YX01 on tobacco growth

**Figure 5:**
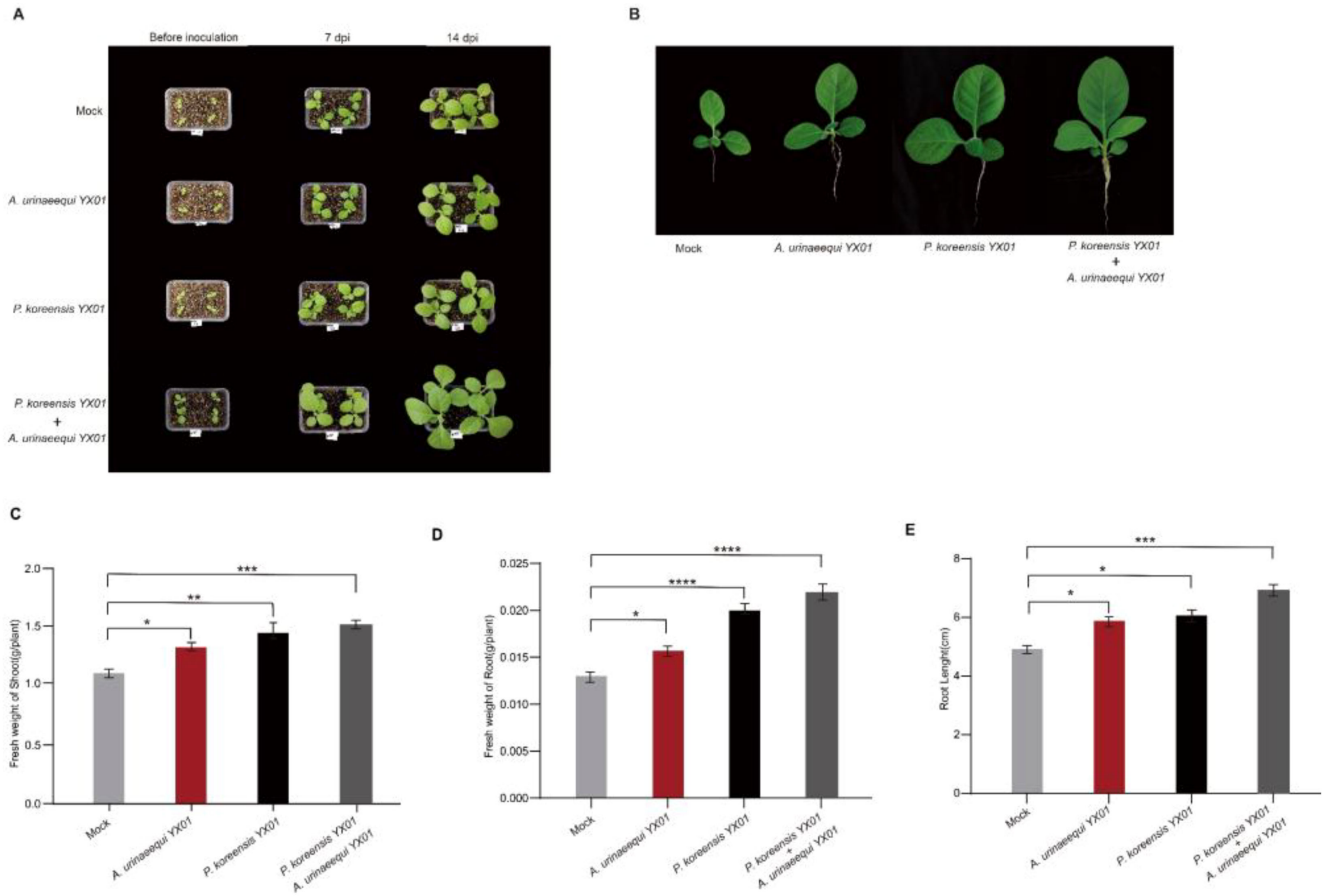
Effects of individual and combined inoculation of *A. urinaeequi* YX01 and *P. koreensis* YX01 on tobacco growth. **(A)** Representative images of tobacco seedlings before inoculation and at 7 and 14 days post-inoculation (dpi) following mock treatment, inoculation with *A. urinaeequi* YX01, inoculation with *P. koreensis* YX01, or co-inoculation with both strains. **(B)** Representative plant phenotypes at 14 dpi showing enhanced shoot and root growth in inoculated plants compared with mock-treated controls. **(C–E)** Quantitative analysis of shoot fresh weight **(C)**, root fresh weight **(D)** and root length **(E)** at 14 dpi. All bacterial treatments significantly promoted tobacco growth relative to the mock control, with *P. koreensis* YX01 and the co-inoculation treatment exhibiting greater growth-promoting effects than *A. urinaeequi* YX01 alone. Values represent means ± SD (n = 4 biological replicates). Statistical significance was determined by one-way ANOVA; asterisks indicate significant differences (*p* < 0.05). The experiment was independently repeated three times with similar results.

To evaluate the growth-promoting effects of *A. urinaeequi* YX01, *P. koreensis* YX01, and their co-inoculation on tobacco, seedlings of cultivar Yunyan 87 were inoculated with the individual strains or both strains together, with mock-inoculated plants serving as controls (**Figure 5A**). Phenotypic observations showed that all bacterial treatments promoted leaf expansion and root development compared with the mock control, with *P. koreensis* YX01 and the co-inoculation treatment exhibiting more pronounced growth-promoting effects (**Figure 5B**).

At 14 dpi, *A. urinaeequi* YX01 increased shoot fresh weight, root fresh weight, and root length by 18.75%, 23.07%, and 19.30%, respectively, relative to the control. The corresponding increases in the *P. koreensis* YX01 treatment were 30.10%, 48.07%, and 21.28%, respectively. Co-inoculation resulted in the highest observed values for all measured growth parameters, increasing shoot fresh weight by 36.93%, root fresh weight by 69.23%, and root length by 37.12% relative to the mock control (**Figures 5C–E**). Statistical analysis confirmed that the co-inoculation treatment significantly improved growth parameters compared with the mock control and showed significantly greater effects than *A. urinaeequi* YX01 alone and *P. koreensis* YX01 alone. Overall, these results demonstrate that both individual strains and their combined inoculation effectively promote tobacco growth and root development, with the co-inoculation treatment showing the strongest growth-promoting effect under the experimental conditions.

## Discussion

This study investigates the molecular function of *NtabCrRLK47*, a RLK gene in tobacco, using CRISPR/Cas9-mediated gene editing. Our findings emphasize the pivotal role of *NtabCrRLK47*-associated microbiome remodeling may contribute to the enhanced growth phenotype. Specifically, we show that the mutation of *NtabCrRLK47* leads to significant alterations in plant growth, including enhanced plant height, root length, leaf area, and an enriched microbial community composition in the rhizosphere. These findings suggest a potential role for microbiome remodeling in plant growth promotion.

Mutations in RLKs genes are known to affect various plant processes, including growth and stress responses (Gandhi and Oelmüller, 2023). In this study, the *NtabCrRLK47* knockout plants showed enhanced root and shoot growth, with notable increases in plant height and leaf area. These results support the hypothesis that *NtabCrRLK47* functions as a negative regulator of plant growth. The enhanced root growth observed in *Ntabcrrlk47* mutants also suggests that this gene plays a role in restricting root elongation under normal conditions, which could be alleviated by the mutation. This aligns with previous research that suggests RLKs mediate root architecture, particularly under stress (Ou et al., 2021). In addition to its role in regulating plant growth, *NtabCrRLK47* also influences the composition of the root-associated microbiota. The CRISPR/Cas9-generated knockout lines provided a model to explore potential shifts in microbial composition that may contribute to the observed growth phenotype. RLKs are known to mediate plant-microbe interactions (Zhao et al., 2020), and our data suggest that *NtabCrRLK47* mutation alters the establishment of the plant’s root microbiome. This alteration could indirectly influence plant growth, as the microbiome plays a crucial role in nutrient acquisition, pathogen defense, and overall plant health (Dwivedi et al., 2025). While further studies are needed to fully elucidate the mechanisms at play, these preliminary findings indicate that *NtabCrRLK47* may shape the root microbiome in a way that enhances plant health and growth.

The mechanisms by which *NtabCrRLK47* regulates rhizosphere microbiome assembly remain to be fully elucidated. However, several processes associated with CrRLK1L family receptor-like kinases may contribute to the observed microbial shifts. CrRLK1L proteins are known to function in cell wall integrity sensing, immune signaling, and the perception of extracellular peptide ligands, thereby coordinating plant growth and environmental responses (Franck et al., 2018; Galindo-Trigo et al., 2016). Disruption of *NtabCrRLK47* may alter root physiological status and defense signaling, leading to changes in the rhizosphere environment that influence microbial recruitment. For example, RLK-mediated signaling has been linked to the regulation of reactive oxygen species production and immune responses, both of which can affect microbial colonization of plant roots (Stegmann et al., 2017; Guo et al., 2018). In addition, alterations in cell wall-associated signaling may modify root–microbe recognition processes and influence the establishment of beneficial microbial populations (Bacete et al., 2018; Engelsdorf et al., 2018). Another possible mechanism involves changes in root exudation patterns. Root exudates serve as important nutritional and signaling molecules that shape rhizosphere microbial communities, and modifications in RLK-regulated developmental or metabolic pathways could indirectly affect the composition of these exudates (Sasse et al., 2018; Pascale et al., 2020). Therefore, the enrichment of beneficial taxa observed in *Ntabcrrlk47* mutants may result from integrated effects on immune regulation, cell wall-associated signaling, and root exudate-mediated microbial selection. Although these mechanisms were not directly examined in the present study, they provide plausible explanations for the observed association between *NtabCrRLK47* disruption and microbiome remodeling and warrant further investigation.

Our findings can also be considered in the context of previous studies on FER, one of the best-characterized members of the CrRLK1L family involved in rhizosphere microbiome regulation. FER has been shown to control the abundance of beneficial *Pseudomonas* species in the rhizosphere through the regulation of basal reactive oxygen species levels and root immune responses, with *fer* mutants exhibiting increased enrichment of *Pseudomonas* populations (Song et al., 2021). Interestingly, although our community-level analysis identified enrichment of *Anaeromyxobacter* and *Entotheonella* as major taxa associated with the *Ntabcrrlk47* mutant, we additionally isolated the growth-promoting strain *P. koreensis* YX01 from the mutant rhizosphere. This observation suggests potential similarities between NtabCrRLK47 and FER in regulating the recruitment of beneficial microbes. Given that both proteins belong to the CrRLK1L family, it is plausible that they share conserved mechanisms involving immune signaling, reactive oxygen species homeostasis, or root surface-associated processes that influence microbial colonization. Nevertheless, the microbial shifts observed in *Ntabcrrlk47* mutants were not identical to those reported for *fer* mutants, indicating that different CrRLK1L family members may exert both conserved and receptor-specific effects on rhizosphere microbiome assembly. Further comparative studies will be required to determine the extent of functional overlap between NtabCrRLK47 and FER in regulating plant–microbe interactions.

Our 16S rRNA sequencing analysis revealed significant changes in the microbial diversity of *Ntabcrrlk47* mutant roots compared to WT roots. Alpha diversity analysis showed a marked increase in microbial diversity in the mutant roots (p < 0.001, Wilcoxon signed-rank test), indicating that the mutation creates a more permissive environment for microbial colonization. These results are consistent with previous studies, which have shown that plant genotype plays a key role in shaping the diversity of root microbiomes (Bulgarelli et al., 2015). Furthermore, beta diversity analysis demonstrated distinct clustering of the microbial communities between mutant and WT roots, suggesting that *NtabCrRLK47* mutation induces significant shifts in microbial composition. These shifts may improve the plant’s interaction with beneficial microbes, which could enhance growth and stress resilience (Edwards et al., 2018; Lai et al., 2025).

At the phylum level, the *Ntabcrrlk47* mutants exhibited an increased abundance of *Myxococcota* and *Entotheonellaeota*, while WT plants were dominated by *Proteobacteria* and *Actinobacteria*. These taxonomic shifts suggest that *NtabCrRLK47* plays a role in regulating the composition of the dominant microbial groups in the rhizosphere. *Myxococcota* and *Entotheonellaeota* are relatively under-studied in plant microbiomes but have been implicated in soil nutrient cycling and organic matter degradation (Li et al., 2023; Naylor et al., 2022). Their increased presence in mutant roots suggests that *NtabCrRLK47* may modulate the microbiome to promote nutrient cycling and potentially improve plant health by fostering beneficial microbes that support plant growth and resistance to pathogens. At the genus level, we observed significant enrichment of *Anaeromyxobacter* and *Entotheonella* in the mutant roots. These genera are known for their beneficial roles in nitrogen cycling and plant defense mechanisms (Zhang et al., 2023; Liu et al., 2016). Their enrichment in the *Ntabcrrlk47* mutants suggests that the mutation might enhance beneficial plant-microbe interactions, improving plant growth, stress resilience and pathogen resistance.

The Gene Ontology (GO) enrichment analysis revealed that the *NtabCrRLK47* mutation significantly reshapes the functional landscape of the tobacco root microbiome. Specifically, we observed enrichment of genes related to biological adhesion, catalytic activity, regulatory functions, and developmental processes (Gu et al., 2024). These findings suggest that the microbiome in *Ntabcrrlk47* mutants is metabolically versatile and better adapted to nutrient acquisition and stress resilience, consistent with previous reports that plants shape their root microbiomes to optimize growth and stress tolerance (Ait-El-Mokhtar et al., 2023; Pascale et al., 2020). KEGG pathway analysis revealed that key metabolic pathways, including carbohydrate metabolism, amino acid metabolism, and energy metabolism, were significantly upregulated in the *Ntabcrrlk47* mutant microbiome. These pathways are critical for microbial survival under fluctuating environmental conditions and for supporting plant nutrition (Abulfaraj et al., 2024). The enrichment of ABC transporters and quorum sensing pathways indicates that the microbiome in *Ntabcrrlk47* mutants has enhanced communication capabilities and increased resilience to environmental stresses (Hartmann et al., 2024; Do et al., 2021). The *NtabCrRLK47* mutation also led to the enrichment of ARGs and multidrug efflux systems, potentially conferring resistance to antimicrobial compounds and enhancing microbial survival in the rhizosphere (Li et al., 2024). The presence of ARGs related to macrolides and monobactams, along with efflux systems, suggests that the *NtabCrRLK47* mutation promotes a microbiome capable of resisting antimicrobial stress, which could provide ecological advantages in soil environments subjected to chemical inputs (Ishikawa et al., 2006). CAZy analysis revealed an enrichment of GHs and GTs in the *Ntabcrrlk47* mutant microbiome. These enzymes are crucial for the degradation of plant-derived carbohydrates and contribute to microbial growth, nutrient cycling, and plant-microbe interactions (Neves et al., 2021; Wang et al., 2013). The enhanced activity of these enzymes suggests that the mutation improves rhizosphere carbon turnover and strengthens the mutualistic interactions between the plant and its microbiome, which could enhance plant stress tolerance and disease resistance (Abulfaraj et al., 2024).

Our results show that the *NtabCrRLK47* mutation favors the recruitment of beneficial microbes like *Anaeromyxobacter* and *Entotheonella*, which enhance tobacco growth. These findings underscore the important role of RLKs in plant-microbe interactions, particularly in regulating microbial recruitment (Galindo-Trigo et al., 2016; Nissen et al., 2016). Similar findings in other species, such as *A. thaliana*, suggest that mutations in RLKs can shift the microbial composition of the rhizosphere towards growth-promoting taxa (Tang et al., 2022; Song et al., 2016). When inoculated together, *Anaeromyxobacter* and *Entotheonella* exhibited synergistic effects, significantly enhancing both shoot and root biomass compared to individual inoculation treatments. This suggests that microbial consortia often outperform single strains in promoting plant growth due to complementary mechanisms and cooperative interactions (Bradáčová et al., 2019; Toju et al., 2018). The combined inoculation of these microbes may optimize nutrient mobilization and promote root system development, resulting in a positive feedback loop that maximizes plant productivity.

Our results underscore the potential of *NtabCrRLK47*-mediated microbiome reprogramming as a strategy for sustainable agriculture. By modulating host genes involved in cell wall sensing and stress signaling, it may be possible to enrich rhizosphere microbiomes with beneficial taxa such as *Anaeromyxobacter* and *Entotheonella*, which individually and synergistically promote plant growth. These findings advance our mechanistic understanding of host-microbiome interactions and open avenues for microbiome-informed strategies in crop improvement, including the development of targeted microbial inoculants for sustainable agriculture.

Although NtabCrRLK47 disruption was associated with distinct shifts in rhizosphere microbial communities and enhanced plant growth, the present study does not establish a direct causal relationship between microbiome remodeling and the growth-promoting phenotype. Future studies employing microbiome transplantation, synthetic microbial communities, or targeted microbial manipulation will be necessary to determine the extent to which microbiome alterations contribute to the observed growth benefits.

## Data Availability

The raw 16S rRNA gene sequencing data have been deposited inthe Genome Sequence Archive (Genomics, Proteomics & Bioinformatics 2025) in National Genomics Data Center (NucleicAcids Res 2026), China National Center for Bioinformation/BeijingInstitute of Genomics, Chinese Academy of Sciences, under accessionnumber CRA036776. The raw metagenomic sequencing data have been deposited inthe same repository under accession number CRA036981. All dataare publicly accessible at https://ngdc.cncb.ac.cn/gsa.

## Supplementary material

Supplementary Figure 1 Comparative analysis of rhizosphere microbial communities in *Ntabcrrlk47* and WT plants. **(A)** Relative abundance of major bacterial phyla in the rhizospheres of *Ntabcrrlk47* and WT plants. WT samples exhibited higher proportions of *Proteobacteria* and *Actinobacteria*, whereas *Ntabcrrlk47* showed increased abundance of *Myxococcota* and *Entotheonellaeota*. **(B)** Relative abundance (%) of *Myxococcota*, which was significantly higher in *Ntabcrrlk47* than in WT. **(C)** Linear discriminant analysis effect size (LEfSe) identifying differentially enriched taxa (LDA score > 2.0) at the phylum, class and family levels, highlighting distinct microbial signatures between *Ntabcrrlk47* and WT. **(D)** Relative abundance (%) of *Entotheonellaeota*, which was significantly higher in *Ntabcrrlk47* than in WT. Data are presented as mean ± SD. **p* < 0.05; **p < 0.01 (Student’s t test).

Supplementary Figure 2 KEGG pathway enrichment heatmap comparing the rhizosphere microbiomes of WT and *Ntabcrrlk47* Plants. Heatmap displaying the relative abundances of selected KEGG pathways in the rhizosphere microbiomes of WT and *Ntabcrrlk47* plants. Pathways associated with biofilm formation, antimicrobial resistance, two-component systems and oxidative phosphorylation were enriched in WT samples, whereas *Ntabcrrlk47* rhizosphere exhibited higher abundances of metabolic pathways, including those involved in fatty acid, amino acid, carbohydrate and cofactor biosynthesis.

Supplementary Figure 3 Differential abundance of glycosyltransferase families in WT and *Ntabcrrlk47* rhizosphere microbiomes. **(A)** Linear discriminant analysis (LDA) scores showing enrichment of the GT4 family in the *Ntabcrrlk47* group. **(B–D)** Boxplots showing the relative abundance of the GT4, GT2_glycos_transf_2 and GT28 families in WT and *Ntabcrrlk47* samples. Statistical significance between groups is indicated by asterisks. **p* < 0.05; ***p* < 0.01 (Student’s t test).

Supplementary Figure 4 Plant growth–promoting effects and molecular identification of bacterial isolates. **(A)** Representative images of tobacco seedlings before inoculation and at 7 and 14 dpi following mock treatment or inoculation with Group #80. **(B)** Comparative growth responses of plants treated with mock, A12 or C8 bacterial isolates, shown before inoculation and at 7 and 14 dpi. **(C)** Representative whole-plant phenotypes showing differential growth promotion by A12 and C8 relative to the mock-treated control. **(D)** Agarose gel electrophoresis of 16S rRNA gene PCR products amplified from *P. koreensis* YX01 isolates. **(E)** Agarose gel electrophoresis of 16S rRNA gene PCR products amplified from *A. urinaeequi* YX01 isolates. **(F)** Sanger sequencing chromatograms and partial 16S rRNA gene sequences confirming the taxonomic identities of *P. koreensis* YX01 and *A. urinaeequi* YX01.

Supplementary Table 1 Sequencing data processing and ASV recovery statistics for *Ntabcrrlk47* mutant and wild-type (WT) tobacco root microbiome samples.

Supplementary Table 2 Isolated microbes from the root sample of *Ntabcrrlk47* mutant.
