# Supplementary material for: Deciphering the role of NtabCrRLK47 in rhizosphere microbiome remodeling and tobacco growth promotion
Source: Front Plant Sci. 2026 Jul 3;17:1864982. doi: 10.3389/fpls.2026.1864982 (PMC13377559; doi:10.3389/fpls.2026.1864982)
Supplement: Supplementary Figure 1 — Comparative analysis of rhizosphere microbial communities in Ntabcrrlk47 and WT plants. (A) Relative abundance of major bacterial phyla in the rhizospheres of Ntabcrrlk47 and WT plants. WT samples exhibited higher proportions of Proteobacteria and Actinobacteria, whereas Ntabcrrlk47 showed increased abundance of Myxococcota and Entotheonellaeota. (B) Relative abundance (%) of Myxococcota, which was significantly higher in Ntabcrrlk47 than in WT. (C) Linear discriminant analysis effect size (LEfSe) identifying differentially enriched taxa (LDA score > 2.0) at the phylum, class and family levels, highlighting distinct microbial signatures between Ntabcrrlk47 and WT. (D) Relative abundance (%) of Entotheonellaeota, which was significantly higher in Ntabcrrlk47 than in WT. Data are presented as mean ± SD. *p < 0.05; **p < 0.01 (Student’s t test). [file DataSheet1.pdf]

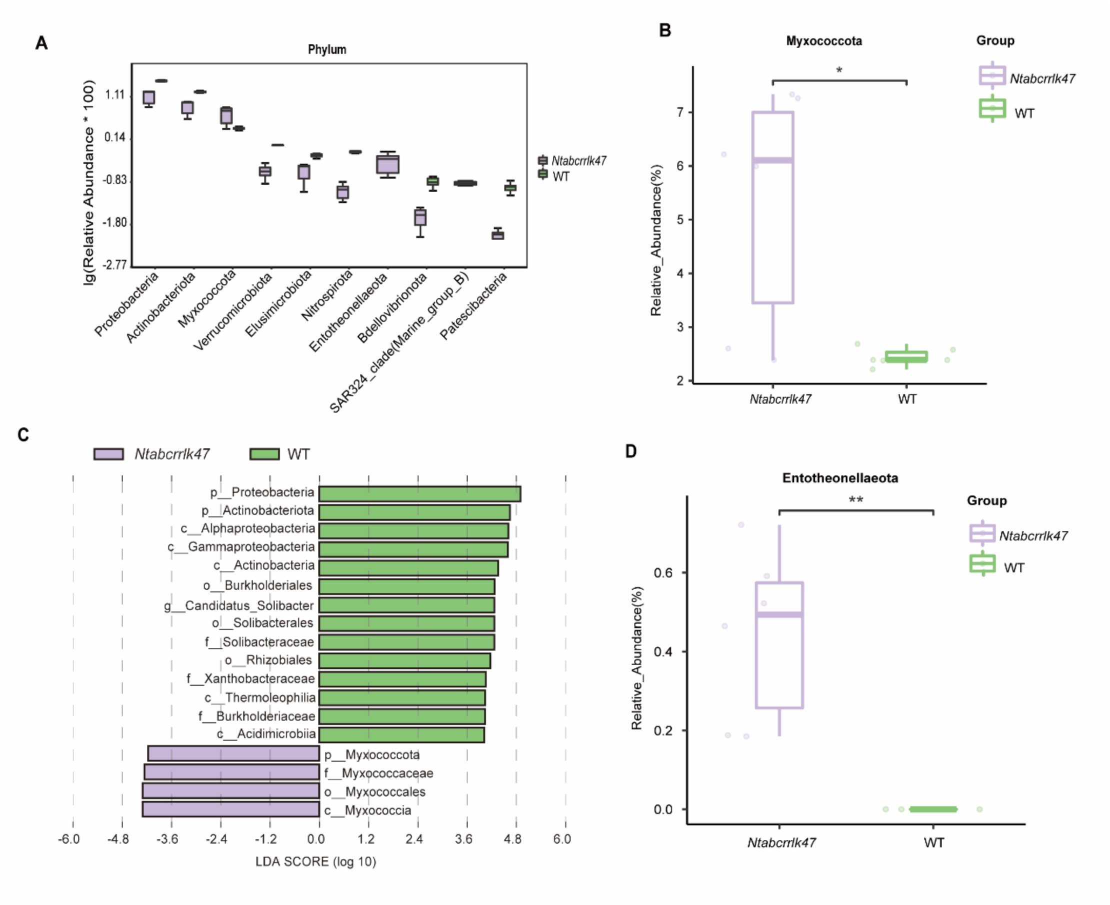

Figure S1. Comparative analysis of rhizosphere microbial communities in *Ntabcrrlk47* and WT plants.

A, Relative abundance of major bacterial phyla in the rhizospheres of *Ntabcrrlk47* and WT plants. WT samples exhibited higher proportions of *Proteobacteria* and *Actinobacteria*, whereas *Ntabcrrlk47* showed increased abundance of *Myxococcota* and *Entotheonellaeota*. **B,** Relative abundance (%) of *Myxococcota*, which was significantly higher in *Ntabcrrlk47* than in WT. **C,** Linear discriminant analysis effect size (LEfSe) identifying differentially enriched taxa (LDA score > 2.0) at the phylum, class and family levels, highlighting distinct microbial signatures between *Ntabcrrlk47* and WT. **D,** Relative abundance (%) of *Entotheonellaeota*, which was significantly higher in *Ntabcrrlk47* than in WT. Data are presented as mean ± SD. *$p < 0.05$; **p < 0.01 (Student's t test).

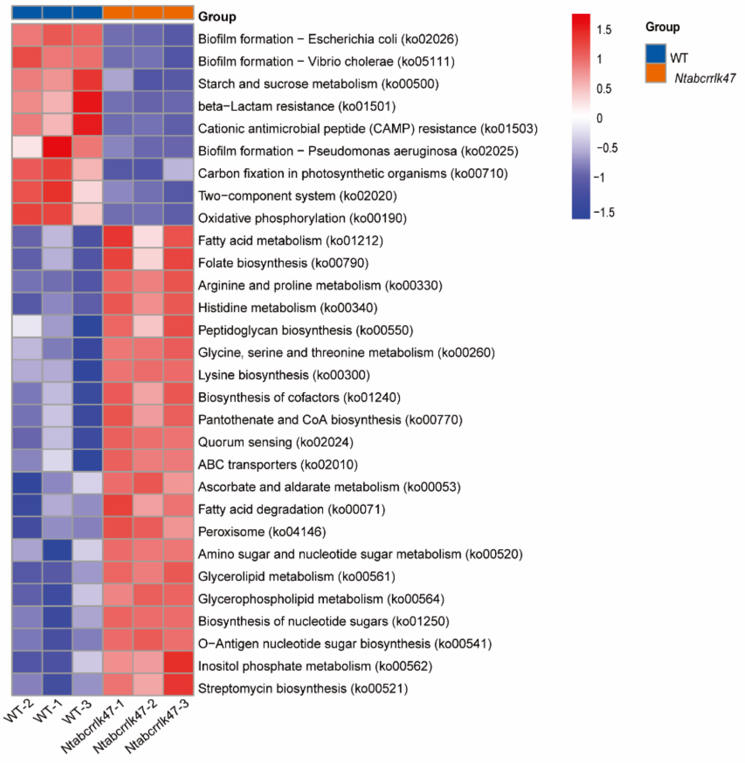

Figure S2. KEGG pathway enrichment heatmap comparing the rhizosphere microbiomes of WT and *Ntabcrrlk47* Plants.

Heatmap displaying the relative abundances of selected KEGG pathways in the rhizosphere microbiomes of WT and *Ntabcrrlk47* plants. Pathways associated with biofilm formation, antimicrobial resistance, two-component systems and oxidative phosphorylation were enriched in WT samples, whereas *Ntabcrrlk47* rhizosphere exhibited higher abundances of metabolic pathways, including those involved in fatty acid, amino acid, carbohydrate and cofactor biosynthesis.

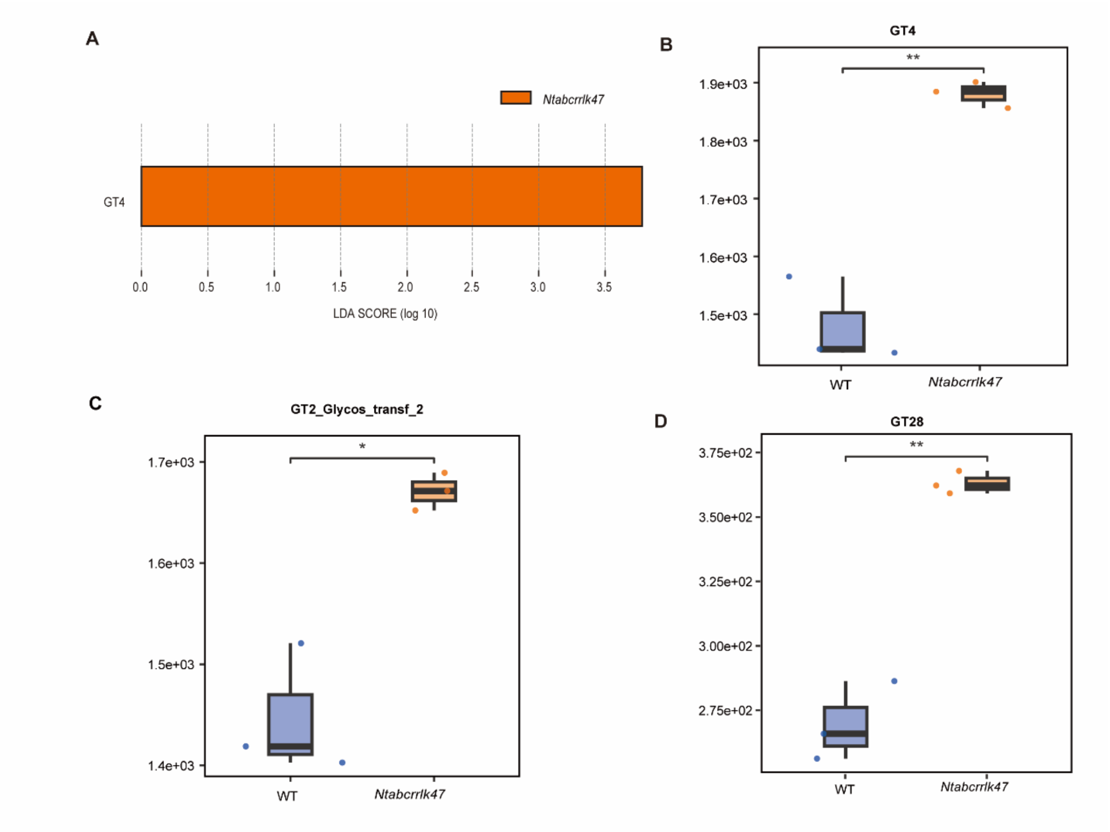

Figure S3. Differential abundance of glycosyltransferase families in WT and *Ntabcrrlk47* rhizosphere microbiomes

A, Linear discriminant analysis (LDA) scores showing enrichment of the GT4 family in the *Ntabcrrlk47* group. **B–D,** Boxplots showing the relative abundance of the GT4, GT2_glycos_transf_2 and GT28 families in WT and *Ntabcrrlk47* samples. Statistical significance between groups is indicated by asterisks. $*p < 0.05$; $**p < 0.01$ (Student's t test).

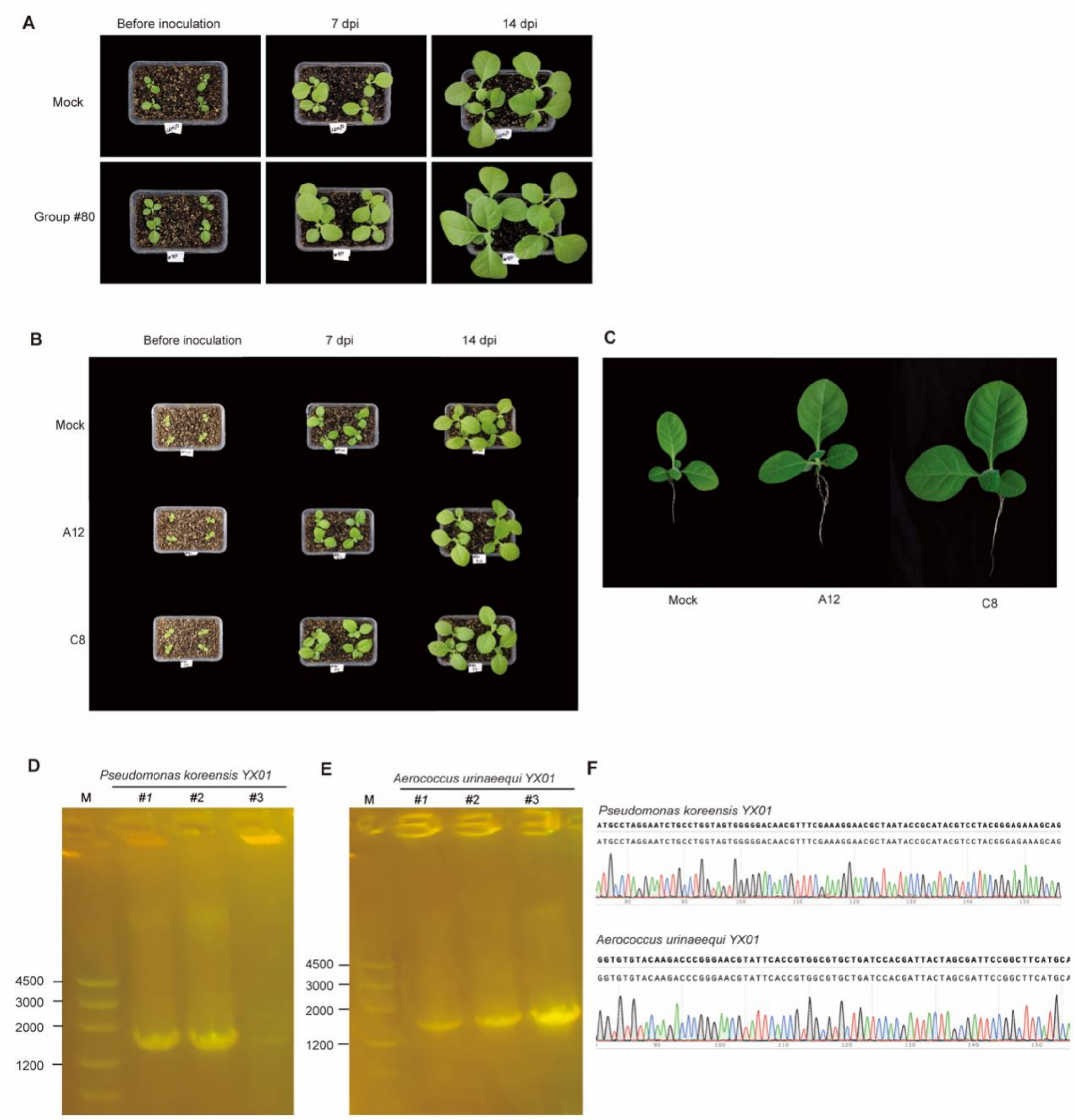

Figure S4. Plant growth–promoting effects and molecular identification of bacterial isolates.

A, Representative images of tobacco seedlings before inoculation and at 7 and 14 dpi following mock treatment or inoculation with Group #80. **B,** Comparative growth responses of plants treated with mock, A12 or C8 bacterial isolates, shown before inoculation and at 7 and 14 dpi. **C,** Representative whole-plant phenotypes showing differential growth promotion by A12 and C8 relative to the mock-treated control. **D,** Agarose gel electrophoresis of 16S rRNA gene PCR products amplified from *P. koreensis* YX01 isolates. **E,** Agarose gel electrophoresis of 16S rRNA gene PCR products amplified from *A. urinaeequi* YX01 isolates. **F,** Sanger sequencing chromatograms and partial 16S rRNA gene sequences confirming the taxonomic

identities of *P. koreensis* YX01 and *A. urinaeequi* YX01.

Table S1: Sequencing data processing and ASV recovery statistics for *Ntabcrrlk47* mutant and wild-type (WT) tobacco root microbiome samples

Sample	Raw Reads	Filtered	Percentage of input passed filter	Denoised	Merged	Percentage of input merged	Non_chimeric	Percentage of input non-chimeric	ASV_counts	Total_ASVs
Ntabcrrlk47-1	78576	74949	95.38	72909	60556	77.07	54934	69.91	679	5230
Ntabcrrlk47-2	79675	76758	96.34	72632	53398	67.02	48633	61.04	835	5230
Ntabcrrlk47-3	78343	74627	95.26	71585	57230	73.05	52085	66.48	1007	5230
Ntabcrrlk47-4	80463	76862	95.52	74791	62137	77.22	57252	71.15	629	5230
Ntabcrrlk47-5	80240	76922	95.86	72555	51969	64.77	47877	59.67	691	5230
Ntabcrrlk47-6	78606	75478	96.02	72392	57878	73.63	53418	67.96	990	5230
WT-1	81312	77855	95.75	74134	56710	69.74	51978	63.92	1222	5230
WT-2	79827	76603	95.96	73429	57074	71.5	52447	65.7	1136	5230
WT-3	80791	77343	95.73	74227	57327	70.96	52636	65.15	1117	5230
WT-4	79021	75876	96.02	72310	55630	70.4	51402	65.05	1151	5230
WT-5	81326	78032	95.95	75086	59850	73.59	55555	68.31	1152	5230
WT-6	79856	76881	96.27	73303	54590	68.36	49623	62.14	1070	5230

Table S2: Isolated microbes from the root sample of *Ntabcrrlk47* mutant

	**Microbes gorups**									
	**#21**	**#22**	**#23**	**#24**	**#25**	**#26**	**#27**	**#28**	**#29**	**#30**
	A1	G7	D10	E3	F8	H1	H11	E6	G1	A2
	G2	H12	A12	C11	H9	G4	G9	C7	A11	A3
**Microbes**	B4	B10	B3	G3	E7	E4	B9	E1	F5	F4
**number**	E5	D8	F7	E10	D1	H4	A10	E2	D7	E8
	D2	G12	H3	D3	H7	D5	G10	B1	C9	G5
									E11	E9
**Gorups**	**#31**	**#32**	**#33**	**#34**	**#35**	**#36**	**#37**	**#38**	**#39**	**#40**
	F7	C11	B12	D2	H11	B5	H5	A12	B4	C9
	D12	A9	B7	A5	H10	H9	A10	C4	C5	G8
**Microbes**	C8	G7	C12	F4	B8	E11	B1	G9	E10	G5
**number**	F10	E8	D8	A6	D4	E3	H2	C6	D3	H1
	D11	B2	F8	C1	E9	A11	C7	B11	D9	B6
							F2			E6
										F11
**Gorups**	**#41**	**#42**	**#43**	**#44**	**#45**	**#46**	**#47**	**#48**	**#49**	**#50**
	D2	C4	B1	A8	C7	F7	G4	F1	D8	B2
**Microbes**	E12	E3	E8	G10	E7	G6	E4	H12	F4	A4
**number**	B12	F10	B9	G11	E5	D1	B10	C8	A9	E6
	D3	C3	F12	E10	F2	H2	E1	H11	G3	B7
						C9	B3	G5	C2	A12
**Gorups**	**#51**	**#52**	**#53**	**#54**	**#55**	**#56**	**#57**	**#58**	**#59**	**#60**
	C7	H4	G12	C3	G1	H2	D7	C5	A2	F1
**Microbes**	G7	F11	F4	H7	A4	C4	E9	D2	H9	G10
**number**	H6	G11	C10	F7	A9	E4	F3	E3	A11	B7
	E8	E1	D4	G4	B6	F12	B4	C9	A3	E6
									A7	D1

										E7
**Gorups**	**#61**	**#62**	**#63**	**#64**	**#65**	**#66**	**#67**	**#68**	**#69**	**#70**
	F12	F4	B9	H7	H12	H5	H11	C8	B6	G1
**Microbes number**	G4	A4	E3	F7	C6	G12	H1	E5	G8	B1
	A1	F2	D3	G6	C9	E4	E12	E1	G5	D12
	F5	D10	A2	H9	D11	D8	E9	H10	F1	D1
							F3	A9	D9	D5
**Gorups**	**#71**	**#72**	**#73**	**#74**	**#75**	**#76**	**#77**	**#78**	**#79**	**#80**
	B7	B5	G11	G4	G10	E9	C6	B2	D9	A3
**Microbes number**	C7	B1	E2	D5	G2	D4	B6	E7	C11	C9
	F12	E5	D11	C4	G6	C2	G1	H10	D2	**C8**
	H7	C5	F4	E10	D7	E12	D1	B11	C12	D8
								A6	A8	**A12**
**Gorups**	**#81**	**#82**	**#83**	**#84**	**#85**	**#86**	**#87**	**#88**	**#89**	**#90**
	H2	A11	H5	H12	A8	A12	B6	C11	C7	D7
	F7	B9	B8	B10	H11	B1	A7	C5	G7	H10
**Microbes number**	G9	D12	H9	E11	E3	F3	B11	A6	C3	F10
	C2	C1	F2	E4	D2	G6	F8	G4	E10	E5
	G10	F9	E12	H6	D9	G1	F4	B3	H4	C6
							B7	D6	D1	B4
**Gorups**	**#91**	**#92**	**#93**	**#94**	**#95**	**#96**	**#97**	**#98**	**#99**	**#100**
	H2	B4	G5	G11	H12	A6	F4	G10	C5	C7
	E8	H5	A8	G2	A12	G3	H11	G9	C3	C9
**Microbes number**	B1	B11	F1	F5	H10	E7	C6	H8	A4	D2
	E6	E2	F2	F12	B6	B2	A7	D10	B9	D7
	D12	B10	E4	H4	A2	H1	G1	D5	A11	H6
										D8
